# Epidemiology and molecular identification of mixed yeast isolates in Malaysia: A way forward

**DOI:** 10.18502/cmm.8.3.11209

**Published:** 2022-09

**Authors:** Humaira Farooq, Tahmina Monowar, Suresh V. Chinni, Swe Swe Latt, Noor Hasliza Zainol, Gokul Shankar Sabesan

**Affiliations:** 1 Faculty of Medicine, AIMST University, Malaysia; 2 Department of Biochemistry, Faculty of Medicine, Bioscience, and Nursing MAHASA University; 3 Faculty of Medicine, RCSI and UCD Malaysia Campus, Penang, Malaysia, Selangor, Malaysia; 4 Pathology Department, Hospital Sultan Abdul Halim, Kedah, Malaysia; 5 Faculty of Medicine, Manipal University College Malaysia, Melaka, Malaysia; 6 Department of Periodontics, Saveetha Dental College and Hospitals, Saveetha Institute of Medical and Technical Sciences, Chennai, India

**Keywords:** *Candida albicans*, *Candida glabrata*, Mixed yeast infections, MspI, PCR-RFLP

## Abstract

**Background and Purpose:**

Invasive candidiasis is one of the most common systemic mycoses, and studies have shown mixed yeast infections. Malaysia lacks mixed yeast culture data.

**Materials and Methods:**

Yeast isolates were collected in Sultan Abdul Halim Hospital, North Malaysia, from October 2020 to October 2021. Chromogenic *Candida* differential agar media and PCR-RFLP were used to identify yeast species.

**Results:**

A total of 206 yeast isolates were collected from different body sites of patients. The majority of the yeast isolates (n=104) were obtained from the urine. Other isolates were extracted from blood (n=52), vaginal swabs (n=45), ear discharge (n=2), tracheal aspirate (n=2), tissue (n=2), skin (n=1), nail (n=1), sputum (n=1), and cerebrospinal fluid (n=1). In total, 200 yeast samples were identified as single species, and six isolates were a mixture of *Candida* species.

**Conclusion:**

Malaysia lacks accurate epidemiological data on mixed yeast infections. We identified all samples to the species level, including mixed yeast cultures, using the *MspI* enzyme and PCR-RFLP.

## Introduction

Fungal infections and antimicrobial drug resistance have become significant threats to public health worldwide, especially in the era of immunosuppression and coronavirus disease 2019 (COVID-19). In most population-based studies [ 1 , 2 ], candidemia is the most common fungal disease among hospitalized patients worldwide. Candidemia is the fourth-to-tenth most common bloodstream infection in hospitalized patients [ 1 - 4 ].

Resistance to the more commonly used antifungal drugs fluconazole and amphotericin B is increasing in many *Candida* species worldwide [ 3 ]. Due to the fact that *Candida* species are becoming more resistant to the most common antifungal drugs, it is important to quickly and accurately identify these pathogens in order to treat them better and avoid problems.

Several studies have reported mixed yeast infections during the last decade. Mycological methods should detect mixed fungal species accurately, especially yeast species with a particular antifungal resistance profile. Among mixed fungemia, it is seen that *C. albicans* is commonly associated with *C. glabrata* and *C. parapsilosis* [ 5 - 7 ].

If a mixed yeast infection remains undetected or is wrongly detected, then the patient may have the risk of being managed by inappropriate antifungal treatment [ 4 ]. Similarly, infections caused by several fungi may be misdiagnosed as monomicrobial illnesses by existing diagnostics, compromising antimicrobial treatment selection and potentially altering clinical outcomes.

Several approaches, including MALDI-TOF, DNA sequencing, and Chromogenic agar, have been used to identify *Candida* species in mixed culture [ 5 , 6 ]. However, they only give probable identification [ 8 ]. In the present study, we identified six mixed yeast infections using the already published PCR-RFLP-based method using the enzyme *MspI*. It is the first study reporting mixed yeast infections in Malaysia using the molecular method.

## Materials and Methods

Yeast isolates were collected in Sultan Abdul Halim Hospital, North Malaysia, from October 2020 to October 2021. The Medical Research and Ethics Committee (MREC), Ministry of Health Malaysia, approved this study (NMRR-20-1588-53243) (IIR).

*C. albicans* (ATCC 10231), *C. parapsilosis* (ATCC 90018), *C. glabrata* (ATCC 15126), and *C. tropicalis* (ATCC 1369) were used as the quality controls. The isolates collected from the hospital were subcultured on Sabouraud dextrose agar (Merck, Germany) and incubated at 30°C for 48 h. Subsequently, the cultures were saved in 80% Glycerol stocks at -80°C. *Candida* Differential Agar Media (Himedia) and PCR-RFLP were used to identify the yeasts.

For molecular identification, DNA was extracted using MasterPure Yeast DNA Purification Kit (Lucigen, USA). Non-enzymatic cell lysis was followed by protein precipitation, nucleic acid precipitation, and resuspension.

The PCR-RFLP was performed based on a standard unique method described by Mirhendi et al. [ 9 , 10 ]. This approach used universal primers, ITS1 (5-TCCGTAGGTGAACCTGCGG-3), and ITS4 (5-TCCTCCGCTTATTGATATGC-3) in PCR to amplify the ITS1-5.8S rRNA ITS2 areas. The final PCR volume was 50 µL. Each reaction comprised 25 µL of master mix, 1 µL of each primer (0.2 M), 3 µL of template, and nucleotide-free water. Conditions for PCR were as follows: initial denaturation at 95°C for 3 min, followed by 35 cycles of denaturation for 30 sec, annealing for 45 sec, and extension for 1 min, with a final extension at 72°C for 5 min [ 9 , 10 ].

Each PCR product was digested with FastDigest *MspI*. (Thermo Fisher Scientific, USA). 10 µL PCR product, 17 µL nuclease-free water, 2 µL 10×buffer, and 1 µL enzyme were incubated at 37°C for 30 min. PCR and RFLP products were separated on a 1.5% agarose gel in TBE buffer for 45 min at 100 V and visualized by staining with ethidium bromide.

Previously reported PCR-RFLP profiles were used to identify yeast species [ 10 , 11 ].

## Results and Discussion

**Figure 1:**
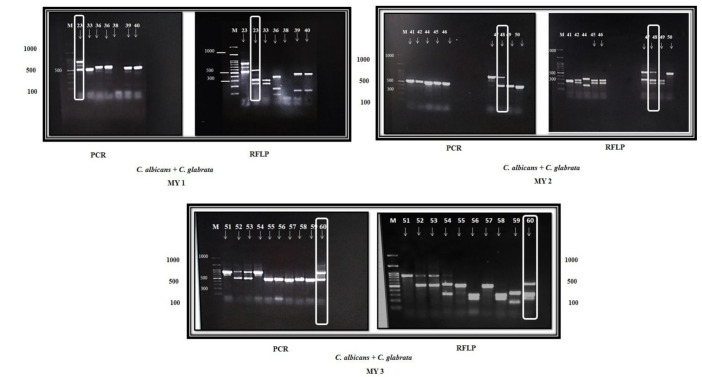
PCR and RFLP band pattern of mixed yeasts (MY): MY1 1 (sample 23), MY 2 (sample 48), mixture 3 (sample 60)

**Figure 2:**
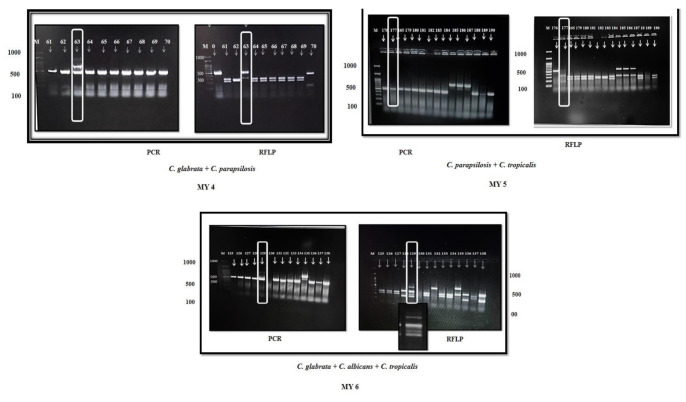
PCR and RFLP band pattern of mixed yeasts (MY): MY 4 (sample 63), MY 5 (sample 177), and MY 6 (sample 129)

A total of 206 yeast isolates were collected from different body sites of patients. The majority of the yeast isolates (n=104) were obtained from the urine. Other isolates were extracted from blood (n=52), vaginal swabs (n=45), ear discharge (n=2), tracheal aspirate (n=2), tissue (n=2), skin (n=1), nail (n=1), sputum (n=1), and cerebrospinal fluid (n=1). Among 206 yeast isolates, 200 isolates were identified as single species, and the most dominant species was *C. albicans*, with a number of 120 (59%). Out of 206 yeast cultures, six isolates were identified as a mixture of different *Candida* species by the PCR-RFLP method. Three mixtures combined *C. albicans* and *C. glabrata* (MY 1, MY 2, and MY 3). The fourth was the mixture of *C. glabrata* and *C. tropicalis* (MY 4), the fifth mixture combined *C. parapsilosis* and *C. tropicalis* (MY 5), and the sixth mixture was the combination of *C. glabrata*, *C. albicans*, and *C. tropicalis* (MY 6) (Figures 1, 2).

**Figure 3:**
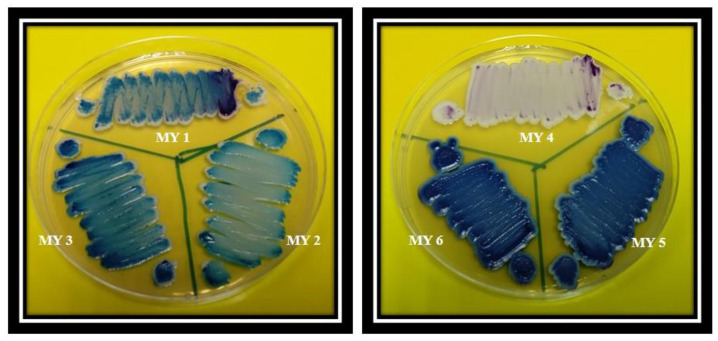
Results of mixed cultures on Chromogenic *Candida* Differential Agar media: MY 1 (sample 23), MY 2 (sample 48), MY 3 (sample 60), MY 4 (sample 63), MY 5 (sample 177), MY 6 (sample 129))

The chromogenic *Candida* differential media results are shown in Figure 3. The results of chromogenic agar only showed a mix of yeasts; however, they could not identify the species, whereas the current PCR-RFLP approach successfully identified the species in mixed cultures.

Classical identification methods cannot identify mixed yeast species due to morphological and phenotypic similarities. These methods are also time-consuming. Researchers tried molecular techniques, such as DNA sequencing and MALDI-TOF, to overcome the difficulties of definitive species identification in mixed cultures; nonetheless, they are not practical or adequate for such infections. The results of this study show that this specific "one enzyme PCR-RFLP" method can be used to find the yeast species in mixed-species cultures.

The ratio of mixed yeast infections (3%) and the combination of species found in this study is the same as reported in previous studies. In most studies, the rate of MY infections was approximately 3% to 4%, while among mixed yeast infections, *C. albicans* is commonly associated with *C. glabrata* as the most popular combination [ 5 - 7 , 12 , 13 ].

In a Taiwanese study, the rate of mixed yeast infections was 3.4% [ 7 ], while in a global multicenter study published in 2021, the overall rate of MY infections was 2.2% [ 6 ]. Many Asian countries, including Thailand and India, took part in this multicenter study, but Malaysia did not. However, in another study, mixed infections were 4% prevalent in Taiwanese hospitals [ 12 ]. The most common combinations in the multicenter study were *C. albicans*/*C. glabrata* (n=42, 34.4%), *C. albicans*/*C. parapsilosis* (n=17, 14%), and *C. glabrata*/*C. tropicalis* (n=8, 6.5%) [ 6 ].

This most common combination of *C. albicans* and *C. glabrata* could be explained by the fact that these two species are the most frequently isolated in epidemiological studies [ 1 ]. Another study found that the hyphal wall adhesins Als1 and Als3 of *C. albicans* play an important role in *C. glabrata* *in vitro* adhesion [ 14 ].

In this study, the chromogenic Candida differential medium could not detect species in mixed cultures. When the quantity of two species in a mixed culture is significantly different, the medium is unable of identifying the minority species [ 7 ].

The "one enzyme PCR-RFLP" molecular approach that was invented by Mirhendi et al. [ 10 ] is an easy molecular method that only requires basic molecular biology equipment, which is usually already present in many diagnostic laboratories [ 15 ]. It is utilized in the identification of yeast and sporulous fungi that are clinically important [ 11 , 16 , 17 ].

## Conclusion

Accurate epidemiology data on mixed yeast infections are unavailable in Malaysia, possibly due to a lack of precise methodological approaches. The molecular method used in this study can identify clinically important yeasts and mixed culture infections. It is inexpensive, quick, and reliable.

## Acknowledgments

This article was published with permission from the Director-General of Health Malaysia.

## Authors’ contribution

H.F. was responsible for study design, development and methodology, collection of data, performing experiments, data analysis, interpretation, writing all/sections of the manuscript, and manuscript revision. T.M. conducted study design, development and methodology, data analysis, interpretation, and manuscript revision. V.S. C. and G.S. were responsible for study design, development and methodology, data analysis, and manuscript revision. S.S.L. conducted data analysis, interpretation, and manuscript revision. N. H. Z. was responsible for the collection of data and manuscript revision. All authors have read and agreed to the published version of the manuscript. 

## Conflicts of interest

There is no conflict of interest.

## Financial disclosure

The university's internal grant for this study's experimental work is appreciated.

## References

[1] Kullberg BJ, Arendrup MC ( 2015). Invasive candidiasis. N Engl J Med.

[2] Antinori S, Milazzo L, Sollima S, Galli M, Corbellino M ( 2016). Candidemia and invasive candidiasis in adults: A narrative review. Eur J Intern Med.

[3] Ranjbar-Mobarake M, Nowroozi J, Badiee P, Mostafavi N, Mohammadi R ( 2021). Cross-sectional study of candidemia from Isfahan, Iran: etiologic agents, predisposing factors, and antifungal susceptibility testing. J Res Med Sci.

[4] Klotz SA, Chasin BS, Powell B, Gaur NK, Lipke PN ( 2007). Polymicrobial bloodstream infections involving Candida species: analysis of patients and review of the literature. Diagn Microbial Infect Dis.

[5] Cassagne C, Normand AC, Bonzon L, 'Ollivier CL, Gautier M, Jeddi F, et al ( 2016). Routine identification and mixed species detection in 6,192 clinical yeast isolates. Med Mycol J.

[6] Medina N, Soto-Debrán JC, Seidel D, Akyar I, Badali H, Baracet A, et al ( 2020). MixInYeast: a multicenter study on mixed yeast infections. J Fungi (Basel).

[7] Yang YL, Chu WL, Lin CC, Tsai SH, Chang TP, Lo HJ ( 2014). An emerging issue of mixed yeast cultures. J Microbiol Immunol Infect.

[8] Nadeem G, Hakim ST, Kazmi SU ( 2010). Use of CHROMagar Candida for the presumptive identification of Candida species directly from clinical specimens in resource-limited settings. Libyan J Med.

[9] Sadrossadati Z, Ghahri M, Imani Fooladi AA, Sayyahfar S, Beyraghi S, Baseri Z ( 2018). Phenotypic and genotypic characterization of Candida species isolated from Candideamia in Iran. Curr Med Mycol.

[10] Mirhendi H, Makimura K, Khoramizadeh M, Yamaguchi H ( 2006). A one-enzyme PCR-RFLP assay for identification of six medically important Candida species. Nihon Ishinkin Gakkai Zasshi.

[11] Mohammadi R, Mirhendi H, Rezaei-Matehkolaei A, Ghahri M, Shidfar MR, Jalalizand N, et al ( 2013). Molecular identification and distribution profile of Candida species isolated from Iranian patients. Med Mycol.

[12] Yang YL, Chu WL, Lin CC, Zhou ZL, Chen PN, Lo HJ, et al ( 2018). Mixed yeast infections in Taiwan. Med Mycol.

[13] Gülmez D, Alp S, Gursoy G, Ayaz CM, Dogan O, Arikan-Akdagli S, et al ( 2020). Mixed fungaemia: an 18-year report from a tertiary-care university hospital and a systematic review. Clin Microbiol Infect.

[14] Tati S, Davidow P, McCall A, Hwang-Wong E, Rojas IG, Cormack B, et al ( 2016). Candida glabrata Binding to Candida albicans Hyphae Enables Its Development in Oropharyngeal Candidiasis. PLOS Pathog.

[15] de Sousa DRT, da Silva Santos CS, Wanke B, da Silva Júnior RM, Cordeiro dos Santos M, Santana Cruz K, et al ( 2015). PCR-RFLP as a useful tool for diagnosis of invasive mycoses in a healthcare facility in the North of Brazil. Electron J Biotechnol.

[16] Mirhendi H, Diba K, Kordbacheh P, Jalalizand N, Makimura K ( 2007). Identification of pathogenic Aspergillus species by a PCR-restriction enzyme method. J Med Microbiol.

[17] Mirhendi H, Makimura K, Zomorodian K, Yamada T, Sugita T, Yamaguchi H ( 2005). A simple PCR-RFLP method for identification and differentiation of 11 Malassezia species. J Microbiol Methods.

